# Supplementary material for: Identification and Validation of Th1-Selective Epitopes Derived from Proteins Overexpressed in Breast Cancer Stem Cells
Source: Vaccines (Basel). 2025 May 15;13(5):525. doi: 10.3390/vaccines13050525 (PMC12115844; doi:10.3390/vaccines13050525)
Supplement: Supplementary file 1 [file vaccines-13-00525-s001.zip › vaccines-3605120-supplementary.pdf]

Table S1. Epitopes used in the screening ELISPOT.

CD105-p95-114	FOXQ1-p183-202
CD105-p116-130	FOXQ1-p205-219
CD105-p214-236	FOXQ1-p271-285
CD105-p258-277	FOXQ1-p370-384
CD105-p302-319	MDM2-p30-49
CD105-p316-335	MDM2-p41-60
CD105-p484-501	MDM2-p57-74
CD105-p569-585	MDM2-p80-96
CD105-p587-611	MDM2-p97-111
CD105-p603-625	MDM2-p104-121
CD105-p626-642	MDM2-p195-211
CDH3-p93-110	MDM2-p460-476
CDH3-p137-156	SOX-2-p45-60
CDH3-p179-197	SOX-2-p91-107
CDH3-p246-264	SOX-2-p193-210
CDH3-p405-425	SOX-2-p217-235
CDH3-p476-493	SOX-2-p267-281
CDH3-p508-525	YB-1-p50-68
CDH3-p601-619	YB-1-p62-81
CDH3-p656-674	YB-1-p82-98
CDH3-p667-684	YB-1-p138-153
CDH3-p778-794	YB-1-p235-249
FOXQ1-p124-144	YB-1-p281-294
FOXQ1-p156-180	

Table S2. Percent mouse homology for the epitopes used in in vivo studies.

Epitope	% Mouse Homology
CD105-p484-501	83
CD105-p569-585	91
CD105-p603-625	95
CD105-p626-642	100
MDM2-p57-74	83
MDM2-p80-96	94
MDM2-p97-111	93
MDM2-p104-121	78
CDH3-p93-110	56
CDH3-p137-156	95
SOX2-p193-210	100
SOX2-p217-235	100
YB1-p138-153	100

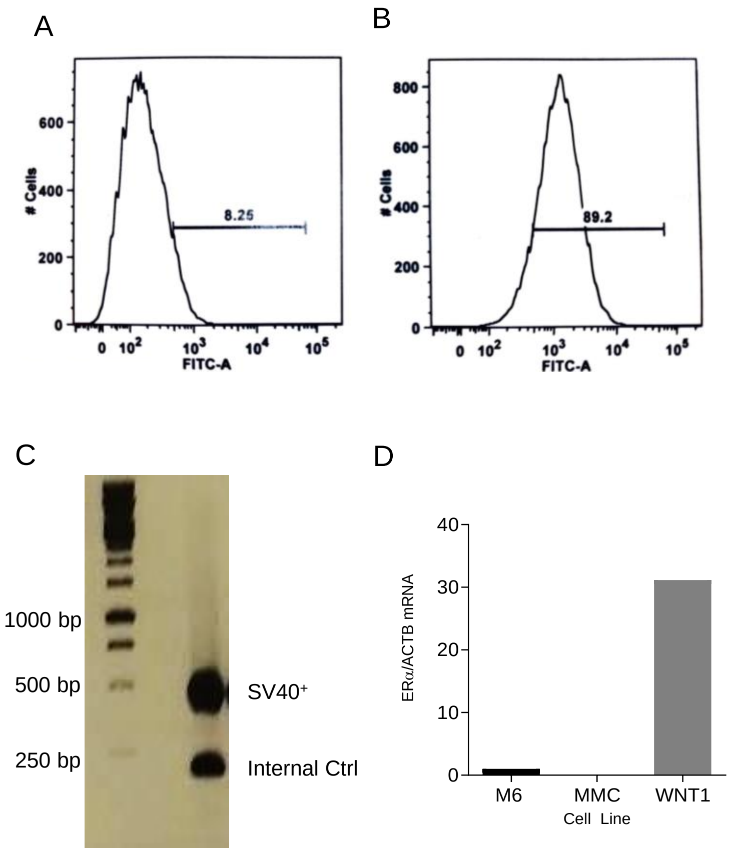

Figure S1. Cell line authentication. Flow cytometric histograms of MMC cells (A) probing with IgG isotype control or (B) anti-HER2/neu. (C) A digital UV-filter photograph of DNA from M6 cells after a PCR using probes specific for the SV40 transgene and an internal control with prominent bands at 500 bp and 200 bp, corresponding to the SV40 transgene and internal control sequence. (D) qRT-PCR for ERα for M6 as well as a negative control (MMC) and positive control (WNT1) cell line.

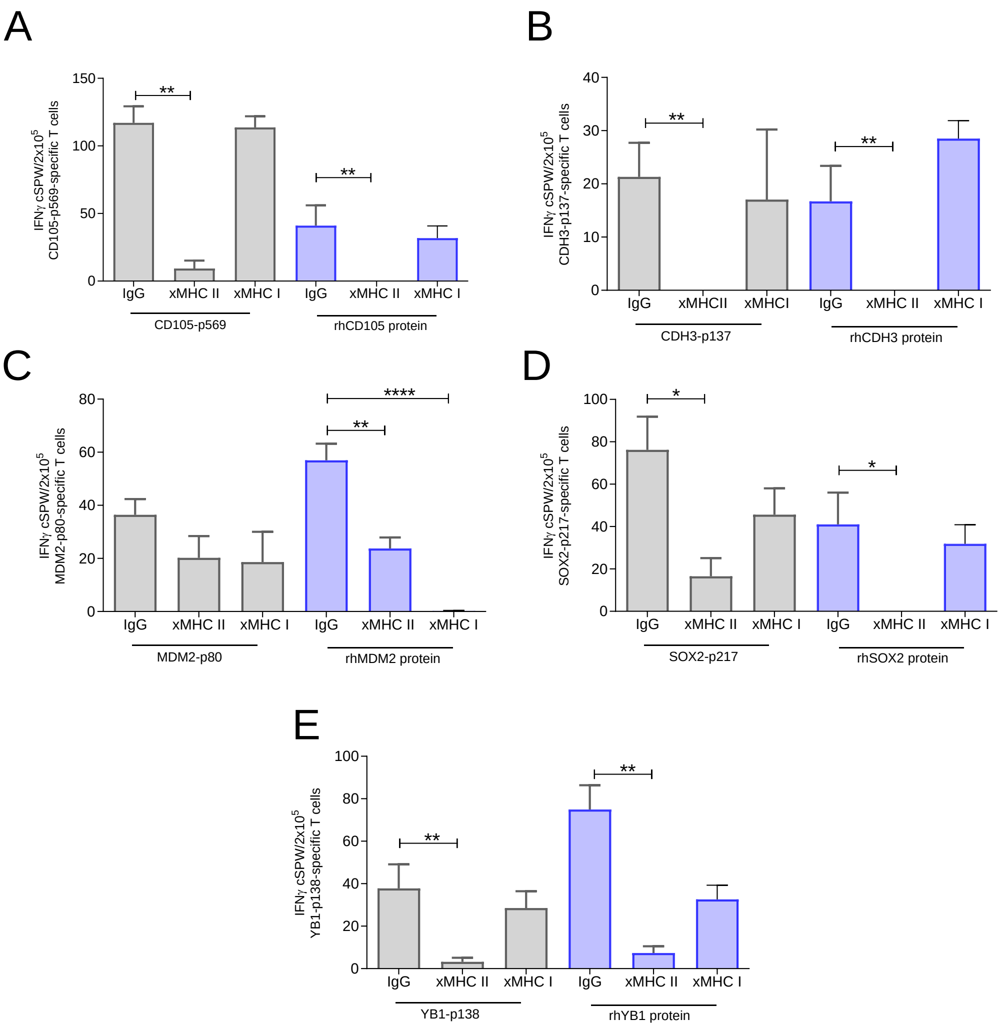

Figure S2. Representative Th1 selective epitopes are MHCII-restricted. Mean (±SEM) IFN-gamma corrected spots per well from peptide specific T-cell lines stimulated with the line generating peptide (gray) or the corresponding recombinant human protein (blue) treated with the indicated antibody for (A) CD105-p569-specific T-cells, (B) CDH3-p137-specific T-cells, (C) MDM2-p80-specific T-cells. (D) SOX2-p217-specific T-cells, (E) YB1-p138-specific T-cells xMHCII=anti-MHCII; xMHCI=anti-MHCI. $*p<0.05$; $**p<0.01$, $****p<0.0001$.
